# Lead Induces Chondrogenesis and Alters Transforming Growth Factor-β and Bone Morphogenetic Protein Signaling in Mesenchymal Cell Populations

**DOI:** 10.1289/ehp.10028

**Published:** 2007-07-03

**Authors:** Michael J. Zuscik, Lin Ma, Taylor Buckley, J. Edward Puzas, Hicham Drissi, Edward M. Schwarz, Regis J. O’Keefe

**Affiliations:** Center for Musculoskeletal Research, University of Rochester School of Medicine and Dentistry, Rochester, New York, USA

**Keywords:** BMP signaling, chondrogenesis, lead, mesenchymal stem cells, TGF-β signaling

## Abstract

**Background:**

It has been established that skeletal growth is stunted in lead-exposed children. Because chondrogenesis is a seminal step during skeletal development, elucidating the impact of Pb on this process is the first step toward understanding the mechanism of Pb toxicity in the skeleton.

**Objectives:**

The aim of this study was to test the hypothesis that Pb alters chondrogenic commitment of mesenchymal cells and to assess the effects of Pb on various signaling pathways.

**Methods:**

We assessed the influence of Pb on chondrogenesis in murine limb bud mesenchymal cells (MSCs) using nodule formation assays and gene analyses. The effects of Pb on transforming growth factor-β (TGF-β) and bone morphogenetic protein (BMP) signaling was studied using luciferase-based reporters and Western analyses, and luciferase-based assays were used to study cyclic adenosine monophosphate response element binding protein (CREB), β-catenin, AP-1, and nuclear factor-kappa B (NF-κB) signaling. We also used an ectopic bone formation assay to determine how Pb affects chondrogenesis *in vivo*.

**Results:**

Pb-exposed MSCs showed enhanced basal and TGF-β/BMP induction of chondrogenesis, evidenced by enhanced nodule formation and up-regulation of *Sox-9*, type 2 collagen, and aggrecan, all key markers of chondrogenesis. We observed enhanced chondrogenesis during ectopic bone formation in mice preexposed to Pb via drinking water. In MSCs, Pb enhanced TGF-β but inhibited BMP-2 signaling, as measured by luciferase reporter assays and Western analyses of Smad phosphorylation. Although Pb had no effect on basal CREB or Wnt/β-catenin pathway activity, it induced NFκB signaling and inhibited AP-1 signaling.

**Conclusions:**

The *in vitro* and *in vivo* induction of chondrogenesis by Pb likely involves modulation and integration of multiple signaling pathways including TGF-β, BMP, AP-1, and NFκB.

In spite of effort to reduce human exposure, lead toxicity continues to be a major environmental health concern in the United States and in other industrial countries. Although recent research has focused on understanding its toxic effects in the developing peripheral and central nervous system, Pb also impacts other developmental processes, including those that occur in the hematologic and skeletal systems (Hicks et al. 1996; Landrigan 1991; Nemoto et al. 2000; Waldron 1979). In fact, the skeleton has long been recognized as a major reservoir for ingested Pb (Barry 1975), and a number of reports demonstrate an inverse correlation between blood Pb concentrations and growth in terms of height, weight, and chest circumference (Schwartz et al. 1986; Shukla et al. 1989, 1991).

Given the strong epidemiologic data, as well as our findings that fracture healing and chondrocyte differentiation are affected by Pb, we have performed a series of *in vitro* experiments to elucidate the underlying mechanism of toxicity during chondrogenesis and chondrocyte differentiation. We employed micromass cultures of mesenchymal stem cells, a culturing technique that has been used previously as a model to study the early events of embryonic limb development (Ahrens et al. 1993). Cell lines with chondrogenic potential, such as C3H10T1/2 cells, and primary limb bud mesenchymal cells plated at high density undergo differentiation to form distinct cartilage nodules. Thus, besides being used to study limb development, these models have been used to define factors and signaling events involved in chondrogenesis (Weston et al. 2000; Weston and Underhill 2000). Transforming growth factor-β (TGF-β) and bone morphogenetic protein (BMP) both induce chondrogenesis in mesenchymal stem cell populations (Chen et al. 1991; Frenz et al. 1994; Iwasaki et al. 1993; Joyce et al. 1990). Prostaglandin E_2_, through protein kinase A signaling also induces chondrogenesis (Biddulph et al. 1988b; Capehart et al. 1990; Clark et al. 2005), whereas retinoic acid is considered an inhibitor of chondrogenic commitment (Biddulph et al. 1988a; Jiang et al. 1995) possibly via down-regulation of TGF-β/Smad signaling (Yu and Xing 2006). Thus various signaling pathways are associated with chondrogenesis, but BMP signaling is particularly important because recombinant BMP proteins are currently approved for clinical use to enhance bone healing in tibia nonunions and spine fusion.

Regarding the signaling pathways, TGF-β and BMP signals are mediated by Smad transcription factors that bind to type I TGF-β receptors and are phosphorylated following lig-and binding to type II receptors (Massague et al. 1997; Mehra and Wrana 2002). Both the BMP receptor–associated Smads (1, 5, and 8) and the TGF-β receptor–associated Smads (2 and 3) are released into the cytoplasm upon phosphorylation, complex with Smad4, and translocate into the nucleus where they regulate gene expression. Because TGF-β receptor–and BMP receptor–associated Smads compete for Smad4 and other downstream signaling molecules, these pathways antagonize one another such that when one pathway increases, the other decreases (Candia et al. 1997). They also have opposing effects in chondrocytes in which TGF-β inhibits (Ballock et al. 1993; Zhang et al. 2004) and BMP promotes (Grimsrud et al. 1999, 2001; Leboy et al. 1997) chondrocyte differentiation. However, in mesenchymal stem cells, both TGF-β and BMP have been shown to enhance chondrogenesis, with the most robust effect in response to BMP signaling (Tuan 2003; Zhang et al. 2004). Assessing the impact of Pb on the propogation of these specific signaling events would provide mechanistic insight into how Pb may affect chondrogenesis and chondrocyte differentiation.

Regarding possible molecular mechanisms of toxicity, Pb has been shown to regulate and alter numerous signaling pathways. Pb has been shown block calcium signaling, inhibit Ca^2+^/phospholipid-dependent protein kinase C (PKC) signaling in neurologic tissues, and interfere with long-term learning and other functions (Pokorski et al. 1999; Vazquez and Pena 2004). Calcium-independent effects include neuronal release of γ-aminobutyric acid (Braga et al. 1999), induction of ERK1/2 (extracellular signal regulated kinase 1 and 2) and p38 (mitogen-activated protein kinase; MAPK) phosphorylation and activation in slices of catfish cerebellum (Leal et al. 2006) and rat hippocampus (Cordova et al. 2004), and activation of ERK1/2 in the GT1-7 neuronal cell line (Zhang et al. 2003) and in lung CL3 cells (Lin et al. 2003). In immortalized human fetal astrocytes, Pb induces vascular endothelial growth factor expression through activation of PKC/AP-1 signaling (Hossain et al. 2000).

*In vitro* studies have shown important Pb effects on chondrocyte phenotype, including decreased proliferation, inhibition of type X collagen expression and synthesis, and an increase in proteoglycan synthesis (Hicks et al. 1996). In addition, Pb has important *in vivo* effects on endochondral bone formation, as Pb-exposed mice and rats have altered growth plate morphology and decreased longitudinal growth (Gonzalez-Riola et al. 1997; Hamilton and O’Flaherty 1994). Correlating with this, we have found that Pb affects developmental/healing processes in the skeleton. This is based on our recent finding that Pb exposure inhibits fracture healing in mice (Carmouche et al. 2005). We have also shown that Pb alters TGF-β and parathyroid hormone–related peptide signaling in chondrocytes undergoing hypertrophic differentiation in culture, an important cellular process that occurs during skeletal development and growth, as well as during healing (Zuscik et al. 2002b). Thus, further elucidation of the molecular mechanism of Pb toxicity during skeletal healing and chondrogenic differentiation in developmental situations is of paramount importance. For the experiments in this study, we used primary limb bud mesenchymal stem cells obtained from stage E11.5 mice, as well as an ectopic bone formation model *in vivo* in mice, to demonstrate a robust chondrogenic stimulation by Pb. Signaling experiments in the cell model indicate that Pb enhances TGF-β signaling and impaires BMP signaling. Interestingly, although Pb increased chondrogenesis on its own in micromass cultures of the limb bud cells, it enhanced the chondrogenic effects of both TGF-β and BMP-2. When considering this finding from the perspective of the signaling data, it is clear that Pb mediates its effects on chondrogenesis in a Smad-independent manner.

## Methods

### Mesenchymal stem cell isolation and culture

We prepared stage E11.5 mouse limb bud mesenchymal stem cells (MSCs) by enzymatic digestion of limb bud tissue as previously described (Zhang et al. 2004). Briefly, CD-1 female mice were euthanized via cervical dislocation 11.5 days after induction of pregnancy, and embryos were removed and placed in Puck’s saline containing glucose. With the aid of a dissecting microscope, forelimbs were removed with forceps and placed in a Puck’s saline solution containing 10% chick serum (Gibco Invitrogen, Carlsbad, CA). Following harvest, limb buds were digested for 45 min in the identical solution supplemented with 0.1% dispase (Sigma, St. Louis, MO). After the digestion, cells were washed several times in serum-free Dulbecco’s modified Eagle medium (DMEM) and counted. Freshly isolated cells were plated in micromass (10^5^ cells/10 μL) with a DME/F12 mixture (40%/60%) supplemented with 10% fetal bovine serum (FBS; Gibco) as previously described (Zhang et al. 2004). One micromass was seeded per well in 24-well plates. After attachment of the cells, wells were flooded with 0.5 mL of the same culture medium mix and experiments commenced.

### Cartilage nodule formation assay in micromass cultures of MSCs

Three days after seeding of micromass cultures and treatment with various Pb concentrations and/or factors of interest, wells were washed once with phosphate-buffered saline (PBS; pH 7.4) and then fixed for 20 min with 1 mL 10% neutral buffered formalin (VWR, Buffalo Grove, IL). The cultures were then washed three times with sterile distilled, deionized water, with the last wash being left on the cells for 15 min. Subsequently, 0.5 mL 3% alcian blue (Sigma) in glacial acetic acid was added to each well for 24 hr at room temperature. After two washes with 70% ethanol and three washes with sterile distilled, deionized H_2_O, photomicrographs of the stained cultures were obtained. Quantification of alcian blue staining was performed using NIH Image (National Institutes of Health, Bethesda, MD). The threshold was standardized to detect nodule area, and the area of the staining was converted from pixels to square millimeters. The area and intensity values were recorded and used to determine staining intensity.

### RNA isolation and real time reverse transcription-polymerase chain reaction (RT-PCR)

Cultures were harvested at various time points, and mRNA was prepared using Trizol reagent (Invitrogen, Carlsbad, CA) as directed by the manufacturer. cDNA was generated using the Advantage RT-for-PCR kit (Clontech, Mountain View, CA), and real-time quantitative RT-PCR was performed as described previously (Zuscik et al. 2002a, 2004). Briefly**,** reactions were carried out using the Rotor Gene Real-Time DNA Amplification System (Corbett Research, Sydney, Australia) according to the manufacturers instructions. cDNA samples were treated with RNase A (Roche Applied Science, Indianapolis, IN), and 50-ng aliquots were combined in a 20-μL final volume with 0.5 mM primers and the SYBR Green PCR Master Mix (Applied Biosystems, Foster City, CA). The PCR protocol began with 94°C denaturation (5 min) and then followed by 35 cycles of the following: 94°C denaturation (20 sec); 50–60°C annealing (20 sec; primer dependent); and 68°C extension (30 sec). Dilutions of the cDNA pool were used for each primer set to generate a standard curve, which was utilized by RotorGene software (Corbett Life Science, Sydney, Australia) to perform quantitative analyses. Data were normalized to the β-actin expression level for each sample. All samples were run in triplicate and repeated four times (*n* = 4).

### *In vivo *chondrogenesis/bone formation assay

Use of mice in this experiment was reviewed and approved by the University of Rochester Institutional Animal Care and Use Committee (IACUC). Mice were treated humanely with regard for alleviation of suffering. At 10 weeks of age, female C57/B6 mice were divided into three groups of 35 mice. Groups were exposed to 0, 55, or 230 ppm Pb (in the form of Pb acetate) in drinking water for 6 weeks before initiation of the ectopic bone formation experiment. Our group previously used an atomic absorption method to establish that these doses of Pb exposure correspond to environmentally relevant whole-blood Pb concentrations of 15 and 40 μg/dL for 55 and 230 ppm, respectively (Carmouche et al. 2005).

Ectopic bone formation was induced in mice by the intramuscular implantation of a gel foam substrate impregnated with cells expressing BMP-2. Specifically, C9 cells derived from murine C3H10T1/2 mesenchymal stem cells, which express BMP-2 under control of a doxycycline-repressible promoter, were cultured in DMEM with 10% FBS, 1% penicillin/streptomycin, 2 mM l-glutamine, and 1 μg/mL doxycycline to repress BMP-2 expression (gift from D. Gazit, Hebrew University-Hadassah Medical and Gene Therapy Center, Jerusalem, Israel). Nine days before implantation, doxycycline was removed from the medium to allow BMP-2 expression, which was confirmed using an ELISA kit (R&D Systems, Minneapolis, MN). As a negative control, wild type C3H10T1/2 cells were cultured in basal medium Eagle (BME) with 10% FBS, 1% penicillin/streptomycin, and 2 mM l-glutamine. Cells from both lines were washed, trypsinized, counted, and resuspended in medium at a concentration of 10^6^ cells/100 μL. Surgifoam (type I collagen sponges; Pharmacia, Piscataway, NJ) was cut into 2 mm × 2 mm × 2 mm segments and soaked in medium, then excess fluid was removed. Aliquots (100 μL) of the cell suspension were micropipetted into each section of gel foam and incubated for 2 hr to allow cells to adhere.

Using an IACUC-approved surgical protocol, we placed C9 cell-impregnated gel foam segments into a muscle pouch in the right quadricep of each mouse; C3H10T1/2-impregnated gel foam was placed in the contralateral limb as a negative control. Ectopic nodules were allowed to develop for 3–28 days after implantation. At each time point, tissue was harvested, fixed in 10% formalin for 3 days, embedded in paraffin, and cut into 5-μm sections. Three sections 30 μm apart were stained with alcian blue and counterstained with alcian blue/hematoxylin. Three representative sections from each sample at day 5 and day 7 were analyzed using Osteometrics software (Osteometrics Inc., Decatur, GA). Cartilage cross-sectional area was measured for each slide and included in statistical analysis (for five nodules, *n* = 15 for statistical purposes).

### Luciferase reporter assays

We performed experiments to assess signaling on various pathways including TGF-β/Smad, BMP/Smad, NF-κB, TOPFLASH (basal c-*fos*-luciferase), AP1, and cyclic adenosine monophosphate response element binding protein (CREB). Reporter and control plasmids for each of these pathways was transfected into MSCs plated as a monolayer, and then cells were trypsinized and replated as a micromass as described above. Thus, MSCs were initially plated in monolayer in 6-cm dishes at a density of 8 × 10^5^ cells per dish. Twelve hours after plating, cells were transfected with either P3TP-luciferase (Luc), 12 × Smad binding element (SBE)-Luc, NF-kB-Luc, TOPFLASH, AP1-Luc, or cAMP-responsive element (CRE)-Luc. These transfections were performed using Superfect (Qiagen, Valencia, CA) according to the manufacturers instructions. The SV40 Renilla-Luc plasmid was co-transfected to facilitate determination of transfection efficiency on a well-by-well basis, as previously described (Ferguson et al. 2000). The DNA:transfection-reagent ratio used for all experiments was 1:3 (weight/volume) with the following amounts of DNA in 0.6 mL medium: Reporter of interest, 2 μg; SV40 Renilla-luc, 10 ng. Eight hours after transfection, cells were trypsinized, counted, and re-plated in micromass culture as described above. Within 12 hr of replating, cells were exposed to various treatments; 48 hr later, cells were lysed and extracts were prepared using the Dual Luciferase Assay System (Promega, Madison, WI), as directed by the manufacturer. An Optocomp luminometer (MGM Instruments, Sparks, NV) was used to measure luminescence in the extracts.

### Western blotting

After rinsing the cell layer with PBS, protein was extracted from micromass cultures using Golden lysis buffer containing protease inhibitor cocktail tablets (Roche Applied Science) as previously described (Samuels et al. 1993). The lysate was centrifuged at 12,000 × *g* and the insoluble material was removed. The protein concentration of the soluble material was determined via the Bradford Kit (Invitrogen). Aliquots of protein extract (25 μg) were separated by SDS-PAGE (10% polyacrylamide) and then transferred to a polyvinylidene fluoride membrane (Schleider and Schuell, Dassel, Germany). The blots were probed overnight at 4°C with the following rabbit anti-mouse polyclonal antibodies: phospho-Smad3 (Cell Signaling Technology, Danvers, MA), phospho-Smad1/5/8 (Cell Signaling Technology), or mouse anti-β-actin monoclonal antibody (Santa Cruz Biotechnology, Santa Cruz, CA). All primary antibodies were applied at a 1:1,000 dilution. Blots were further incubated for 1 hr at 20°C in the presence of horseradish peroxidase–conjugated secondary antibodies against rabbit or mouse (both from BioRad, Hercules, CA) at a dilution of 1:3,000. The immune complexes were detected using ECL-Femto (Pierce, Rockford, IL) and visualized via exposure of X-OMAT AR film (Kodak, Rochester, NY).

## Results

### Pb stimulates chondrogenesis and enhances the chondrogenic effect of BMP-2 and TGF-β

**Figure 1:**
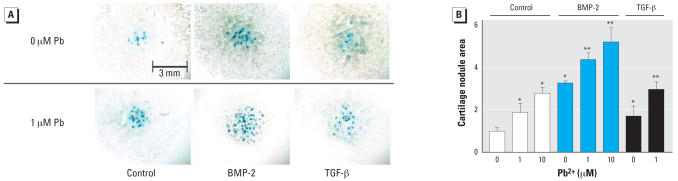
Effects of Pb exposure on chondrogenesis and the chondrogenic impact of BMP-2 and TGF*-*β in MSCs treated with 0, 1, or 10 mM lead acetate either alone or in the presence of 50 ng/mL BMP-2 or 5 ng/mL TGF-β (see “Materials and Methods” for details). (*A*) Representative photomicrographs of stained cultures. (*B*) Quantification of the nodule area (mean ± SE; relative to control). Significance was assessed via ANOVA (*n* = 3; *p* < 0.05). *Significantly different from the untreated control. **Significantly different from either the BMP-2-treated control or the TGF-β-treated control.

MSCs were placed in micromass culture and were treated with Pb in the presence and absence of BMP-2 (50 ng/mL) and TGF-β (5 ng/mL). After 3 days in culture, treatment with Pb resulted in a stimulation of chondrogenesis as measured by the presence of alcian blue nodules (Figure 1A). The effect was dose dependent with quantification of nodule formation showing a 2-fold increase present at 1 μM Pb and nearly a 3-fold increase noted at 10 μM Pb (Figure 1B). As anticipated, treatment of the cultures with BMP-2 or TGF-β led to an induction of chondrogenesis. Although the effect of BMP-2 was slightly greater (3.2-fold) than that of TGF-β (1.7-fold), the induction of chondrogenesis by both growth factors was enhanced by Pb (Figure 1B). In the presence of 10 μM Pb, BMP-2 induced chondrogenesis 5-fold and TGF-β induced a 3-fold increase (Figure 1B). Thus, Pb induces chondrogenesis and enhances the effects of both BMP-2 and TGF-β on this process.

### Pb induces* Sox9*,* col2*, and aggrecan expression and influences regulation of these genes by BMP-2 and TGF-β

**Figure 2:**
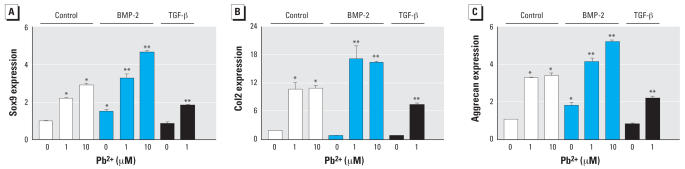
Effects of Pb exposure on Sox9 (*A*), col2 (*B*), and aggrecan (*C*) mRNA expression (mean ± SE; relative to control) in MSCs treated with 0, 1, or 10 μM Pb acetate either alone or in the presence of 50 ng/mL BMP-2 or 5 ng/mL TGF-β. Significance was assessed via ANOVA (*n* = 3; *p* < 0.05). *Significantly different from the untreated control. **Significantly different from either the BMP-2–treated control or the TGF-β–treated control.

To assess chondrogenesis in MSC cultures on the molecular level, the mRNA expression of *Sox9*, *col2*, and aggrecan were determined via real time RT-PCR. Consistent with the alcian blue staining shown in Figure 1, Pb dose-dependently enhanced the expression of these genes, with 10-μM Pb inducing a 2-fold increase in *Sox9*, a 5-fold increase in *col2*, and a 2.5-fold increase in aggrecan compared with untreated cultures (Figure 2). Also consistent with the alcian blue results, Pb enhanced both BMP-2 and TGF-β regulation of these genes. Specifically, BMP-2 induction of *Sox9* and aggrecan was enhanced 2- to 3-fold by 1 and 10 μM Pb (Figure 2A,C). Furthermore, in the presence of both BMP-2 and Pb, *col2* expression was increased to levels above that seen with either BMP-2 or Pb alone (Figure 2B). Comparatively, treatment with a combination of TGF-β and Pb caused an induction of *Sox9*, *col2*, and aggrecan that was generally less than the respective effect of Pb alone (Figure 2A–2C). These results further support the hypothesis that Pb influences chondrogenesis directly and influences chondrogenic regulation induced by BMP-2 and TGF β.

### Pb stimulates BMP-2–induced chondrogenesis* in vivo*

**Figure 3:**
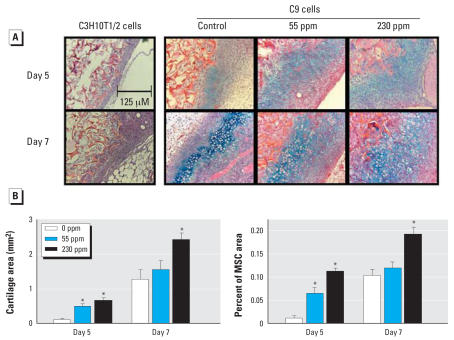
Effects of Pb exposure (0, 55, or 350 ppm) on chondrogenesis in an *in vivo* model [C3H10T1/2 cells or C9 cells (BMP-2 expressing C3H10T1/2 cells)] of ectopic bone formation (see “Materials and Methods” for details). (*A*) Representative photomicrographs of tissues stained with Alcian blue and hematoxylin. (*B*) The cartilage area and the percentage of mesenchyme consisting of cartilage quantified by histomorphometry using Osteometrics software (mean ± SE). Significance was assessed using ANOVA (*n* = 15; *p* < 0.05). *Significantly different from the untreated control at the same time point.

To complement the *in vitro* findings discussed above, chondrogenesis was examined in the context of an *in vivo* murine model of ectopic bone formation in which we used control C3H10T1/2 cells or C9 cells. A total of 1 × 10^6^ cells were loaded into 2 mm × 2 mm × 2 mm type I collagen sponges and then implanted into the medial thigh musculature of C57/B6 mice pretreated with normal drinking water or water containing Pb (55 or 230 ppm) for 6 weeks. In control C3H10T1/2 cell implanted limbs, we observed proliferation of host mesenchymal cells at the periphery of the implant, but no cartilage tissue formed (Figure 3A). This is consistent with the requirement that BMP-2 is required to initiate the chondrogenic commitment of implanted and/or host cells. It should be noted that control cells did not support a chondrogenic response in Pb-exposed mice (data not shown). In limbs implanted with BMP-2–expressing C9 cells, the mesenchymal cell proliferation was associated with evidence of chondrogenesis beginning at 5 days (Figure 3A). Administration of Pb in the drinking water was associated with a dose-dependent increase in chondrogenesis in 5-day and 7-day tissue samples from C9-injected mice. Quantification of the cartilage content via histomorphometry at these time points confirms the dose dependency of the Pb effect, with the 230-ppm Pb level showing the most robust increase in cartilage area and the percent of the mesenchyme that consisted of cartilage (Figure 3B). These findings are consistent with the *in vitro* data presented in Figure 1 by demonstrating that Pb enhances BMP-2 induction of chondrogenic responses.

### Pb increases TGF-β–mediated stimulation of p3TP-Luc and increases Smad3 phosphorylation

**Figure 4:**
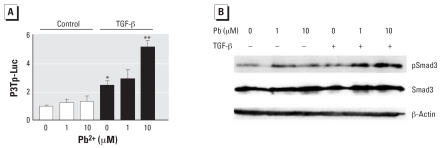
Effects of Pb on TGF-β–induced signaling in MSCs. (*A*) TGF-β–mediated stimulation of p3TP-Luc (mean ± SE; relative to control) in MSCs plated in a monolayer and co-transfected with P3TP-Luc and SV40 Renilla (efficiency control). After 8 hr, cells were trypsinized, counted, and replated in micromass culture; 16 hr after seeding the micromasses, cells were treated with 0, 1, or 10 μM Pb acetate alone or in the presence of 5 ng/mL TGF-β. Forty-eight hours later, the cell layer was solubilized with lysis buffer and sample luminescence was measured; significance was assessed using ANOVA (*n* = 3; *p* < 0.05). (*B*) Representative Western blots of MSCs seeded into micromass culture and treated with 0, 1, or 10 μM Pb acetate 24 hr later. After a 24 hr preexposure period, cultures were treated either with or without 5 ng/mL TGF-β. After 30 min, total cellular proteins were harvested and Western blot analysis was performed to detect phospho-Smad3, Smad3, and β-actin (load control). Blots are from a series of three separate experiments. *Significantly different from the untreated control. **Significantly different from the TGF-β-treated control.

MSCs were transfected with the TGF-β/Smad3 responsive reporter p3TP-Luc and treated with Pb in the presence and absence of TGF-β. Alone, Pb did not alter reporter activity, whereas TGF-β (5 ng/mL) induced a 2.5-fold stimulation (Figure 4A). However, Pb enhanced the effect of TGF-β on the p3TP-Luc promoter, with a maximal 5-fold effect occurring at the 10-μM Pb concentration (Figure 4A). Consistent with these findings, Western blot analysis showed that Pb did not alter levels of phospho-Smad3 alone, but resulted in increased Smad3 phosphorylation in cells stimulated with TGF-β for 1 hr (Figure 4B). These results demonstrate that Pb influences TGF-β signaling and targets Smad3 signaling events.

### Pb inhibits BMP-2-induction of 12 × SBE-Luc and phosphorylation of Smad1/5/8

**Figure 5:**
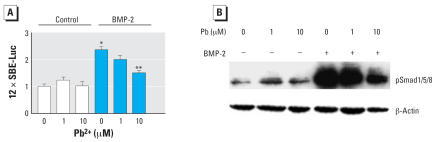
Effects of Pb on BMP-2–induced signaling (*A*) and phosphorylation of Smad1/5/8 (*B*) in MSCs. (*A*) 12 × SBE-Luc signaling (mean ± SE; relative to control) in MSCs plated in a monolayer and co-transfected with 12 × SBE-Luc and SV40 Renilla (efficiency control); after 8 hr, cells were trypsinized, counted, and replated in micromass culture. Sixteen hours after seeding the micromasses, cells were treated with 0, 1, or 10 μM Pb acetate alone or in the presence of 50 ng/mL BMP-2; 48 hr later, the cell layer was solubilized with lysis buffer and sample luminescence was measured. Significance was assessed using ANOVA (*n* = 3; *p* < 0.05). (*B*) Representative Western blot of MSCs seeded into micromass culture and treated with 0, 1, or 10 μM Pb acetate 24 hr later. After a 24-hr preexposure period, cultures were treated either with or without 50 ng/mL BMP-2. After 30 min, total cellular proteins were harvested and Western blot analysis was performed to detect phospho-Smad1/5/8 and β-actin (load control). Blots are from a series of three separate experiments. *Significantly different from the untreated control. **Significantly different from the TGF-β-treated control.

MSCs were transfected with the BMP-responsive reporter 12 × SBE-Luc and treated with Pb in the presence and absence of BMP-2 (100 ng/mL). Pb itself did not alter reporter activity, but BMP-2 stimulated a 2.4-fold induction. Interestingly, Pb induced a dose-dependent inhibition of BMP-2–induced signaling on the 12 × SBE-Luc promoter, with a maximal 65% inhibition occurring at a concentration of 10 μM Pb (Figure 5A). Consistent with this finding, Pb did not alter levels of phospho-Smad1/5/8 alone, although it had a marked dose-dependent inhibitory effect on BMP-2 stimulation of Smad1/5/8 phosphorylation (Figure 5B). Effects were present by 30 min after exposure and were sustained at 1 hr. These results demonstrate that Pb strongly inhibits BMP-2 signaling (which is opposite the effect of Pb on TGF-β signaling) and suggest another mechanism by which Pb can affect the process of chondrogenesis.

### Pb has differential effects on AP-1, CRE, NFκB, and Wnt/β-catenin signaling

**Figure 6:**
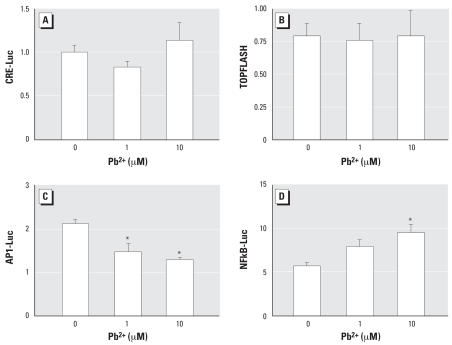
Effect of Pb on MSCs transfected with CRE-Luc (*A*), TOPFLASH (*B*), AP1-Luc (*C*), or NFκB-Luc (*D*). All cultures were co-transfected with SV40 Renilla to control for transfection efficiency. After 8 hr, cells were trypsinized, counted, and replated in micromass culture; cells were treated with 0, 1, or 10 μM Pb acetate 16 hr later. After 48 hr, the cell layer was solubilized with lysis buffer and sample luminescence was measured to determine basal signaling activity on each pathway of interest. Values are mean ± SE normalized to Renilla-Luc. Significance was assessed using ANOVA (*n* = 3; *p* < 0.05). *Significantly different from the untreated control.

Because Pb has no effect on basal TGF-β–Smad and BMP-Smad signaling, and has opposite effects on these signaling pathways in the presence of their respective growth factors, it is unlikely that the induction of chondrogenesis by Pb is related to direct regulation of Smad signaling. To determine the possible involvement of other pathways known to be important in the process of chondrogenesis, MSCs were transfected with AP1-Luc, NFκB-Luc, CRE-Luc, and TOPFLASH reporters, each containing multiple repeats of the respective consensus binding sites. Although there was no effect of Pb on CRE or TOPFLASH activity (Figure 6A,B), Pb caused a dose-dependent decrease in basal AP1 activity that was maximal (40%) in cultures treated with 10 μM Pb (Figure 6C). Finally, Pb induced a dose-dependent but not significant increase in NFκB activity (Figure 6D). These results suggest that influences of Pb on chondrogenesis may also be partially dependent on a direct alteration of AP1 signaling.

## Discussion

There is growing awareness of the pleiotropic effects of Pb, with targets of toxicity including multiple tissues that include the skeleton. In fact, at concentrations considered subtoxic, Pb is known to affect skeletal growth. The two most recent National Health and Nutrition Examination Surveys (NHANES II and NHANES III) have demonstrated that Pb exposure reduces skeletal growth independent of factors such as nutritional status, social/ economic class, and co-morbid diseases (Ballew et al. 1999). Moreover, significant developmental effects are apparent at blood concentrations as low as 4 μg/dl (Ballew et al. 1999). Thus, skeletal tissues, and particularly the growth plate, are among the most sensitive tissue targets of Pb. Representing a first step toward understanding the mechanism underlying the influence of Pb on the skeleton, findings presented in this article demonstrate that Pb targets mesenchymal stem cells and affects the determination of cell fate. Specifically, embryonic limb bud cells exposed to Pb display a severalfold increase in chondrogenenic commitment. Moreover, Pb induces the chondrogenic effects of both BMP-2 and TGF-β. Because embryonic limb bud stem cells also have the potential to differentiate into other mesenchymal tissues, Pb possibly also influences the cell fate on other cell differentiation pathways.

Pb has broad and complex effects on multiple intracellular signaling pathways. As mentioned above, Pb has been shown in various tissues to *a*) block calcium signaling and inhibit Ca^2+^/phospholipid-dependent PKC signaling (Pokorski et al. 1999; Vazquez and Pena 2004); *b*) induce ERK1/2 and p38 (MAPK) phosphorylation and activation (Lin et al. 2003; Zhang et al. 2003); and *c*) activate AP-1 signaling (Hossain et al. 2000). We have found that Pb affects neither basal BMP-2 nor TGF-β signaling; thus it is unlikely that the effect of Pb on chondrogenesis is related to a direct alteration of these signaling pathways. Moreover, although Pb enhanced the effect of BMP-2 on chondrogenesis, it acted as an inhibitor of Smad1/5/8 signaling. Further experiments demonstrated that Pb also regulates other signaling pathways, including AP-1 and possibly NFκB. Thus, it appears that the effects of induction of chondrogenesis and enhancement of TGF-β and BMP-2 on chondrogenesis are secondary to stimulation of other interacting signaling pathways.

Previous work has established that Pb regulates BMP and TGF-β signaling pathways. Oral administration of Pb chloride has been shown to cause a reduction in the expression of TGF-β in intestinal tissues in diabetes-prone NOD mice (Goebel et al. 1999). In an earlier study using chicken growth plate chondrocyte cultures (Zuscik et al. 2002b), we found that both Pb and TGF-β independently inhibit expression of the maturational marker *colX*, Interestingly, this study also showed that inhibition of *colX* by TGF-β was completely reversed by Pb. Furthermore, although neither Pb nor TGF-β alone affected the expression of BMP-6, in combination they induced its expression 3-fold (Zuscik et al. 2002b). Although these previous studies did not fully examine regulation of downstream signaling events, they did demonstrate a regulation of TGF-β signaling. When considered along with the current findings, it seems clear that the influence of Pb on chondrogenesis/chondrocyte differentiation is at least partially dependent upon the presence of TGF-β and activation of its Smad signaling pathway. Overall, our findings suggest that Pb exposure renders mesenchymal precursor cells more sensitive to TGF-β by increasing Smad3 phosphorylation and signaling. To our knowledge, this is the first demonstration of regulation of Smad phosphorylation by Pb in any cell system.

In the present study, we found that BMP signaling is also regulated by Pb. BMP receptor signaling occurs in a manner analagous to the TGF-β pathway, with Smad1/5/8 binding to the type I BMP receptor, followed by phosphorylation of these factors upon ligand activation (Miyazono et al. 2005). Using a polyclonal antibody that recognizes all three BMP receptor–associated Smads, we found that the basal phosphorylation state was not altered by Pb alone, but the induction of phosphorylation of Smad1/5/8 was markedly inhibited by Pb. Similarly, although Pb did not alter basal activation of the BMP-Smad responsive reporter 12 × SBE, Pb significantly reduced activation of this reporter by BMP-2. Thus, similar to TGF-β signaling, Pb regulates BMP signaling only during activation of the pathway by lig-and. Interestingly, the effects are opposite, with Pb inhibiting BMP-Smad phosphorylation and stimulating TGF-β-Smad phosphorylation. Overall, the potent inhibition of BMP-2–induced Smad1/5/8 signaling by Pb represents the most robust signaling effect identified to date in a skeletal cell type with regard to a candidate mechanism of Pb toxicity.

The complex regulation of the Smad signaling molecules makes it unlikely that the induction of chondrogenesis by Pb is cause by direct alteration of these pathways. Although BMP and TGF-β signaling pathways have antagonistic effects on some cells, including growth plate chondrocytes where TGF-β inhibits and BMP-2 stimulates maturation, both signals enhance chondrogenesis in mesenchymal stem cell populations (Hoffmann and Gross 2001). An earlier study has shown that BMP-4 stimulates chondrogenesis in C3H10T1/2 and MC615 chondroprogenitor cells through activation of the BMP-receptor–associated Smads (Hatakeyama et al. 2003). TGF-β signaling through Smad2 and Smad3 is associated with enhanced chondrogenesis in murine mesenchymal stem cells (Yu and Xing 2006). Additionally, the action of these factors is dependent on complex signaling, because it is clear that TGF-β and BMP signals act in combination with other signaling pathways. In ATDC5 cells, stimulation of chondrogenesis by TGF-β is mediated by activation of Smad pathways and the p38 and Erk1/2 MAP kinase pathways simultaneously (Watanabe et al. 2001). Furthermore, whereas Wnt/β-catenin signaling *in vivo* appears to stimulate osteogenesis over chondrogenesis (Day et al. 2005), in human mesenchymal stem cell cultures TGF-β acts synergistically with Wnt/β-catenin signaling to induce chondrogenesis (Zhou et al. 2004). Similarly, BMPs have been shown to act synergistically with Wnt/β-catenin signaling to induce chondrogenesis in C3H10T1/2 cells (Fischer et al. 2002). Overall, it is clear that BMP and TGF-β Smad signaling is critical for induction of chondrogenesis; however, these pathways are only a part of multiple signaling events that contribute to the regulation of chondrogenic commitment.

To extend these signaling results, we also examined the effect of Pb on other signaling pathways. Although no effects were observed on CRE and TOPFLASH activation using the respective luciferase-based reporters, the experiments showed that Pb inhibited basal AP-1-Luc reporter activity. AP-1 activation has been shown to inhibit chondrogenesis, so it is possible that the inhibition of AP-1 could be involved in the induction of chondrogenesis by Pb (Hwang et al. 2005; Tufan et al. 2002). The inhibition of AP-1 signaling in mesenchymal stem cells is in contrast to the findings in other cells where Pb induces AP-1 signaling activity. In a previous study (Zuscik et al. 2002b), we showed that Pb increased AP-1 and NFκB signaling in chick embryonic chondrocytes in culture. Furthermore, *in vitro* Pb exposure in rats results in activation of AP-1 and NFκB levels in multiple regions of the brain and in astrocytes in culture (Ramesh et al. 2001). *In vivo* Pb exposure results in increased NFκB signaling in renal tubular cells and results in nephritis in rats (Rodriguez-Iturbe et al. 2005). Similarly we found that Pb induces NFκB signaling in MSCs as measured by induction of NFκB-Luc reporter. However, because signaling on the NFκB pathway has been shown to destabilize Sox9 mRNA and inhibit chondrogenesis (Sitcheran et al. 2003), it is unlikely that Pb-induction of NFκB signaling seen in MSCs is directly causing an enhanced chondrogenic response.

The induction of chondrogenesis by Pb in the current study is consistent with findings observed in an *in vivo* murine model of fracture healing (Carmouche et al. 2005). Mice with Pb levels similar to those found in humans with Pb intoxication had delayed healing of stabilized femur fractures. The effect was dose dependent, and cartilage was observed to be a major target. Pb-exposed mice had increased cartilage volumes, delayed chondrocyte maturation, persistence of cartilage, and reduced bone formation (Carmouche et al. 2005). Overall, when considered along with the results of Carmouche et al. (2005), our findings in the present study further support the hypothesis that Pb is an inducer of chondrogenesis.

In conclusion, the present study establishes that in addition to affecting chondrocyte maturation, Pb accelerates the differentiation of mesenchymal precursors into chondrocytes. Although Pb alters BMP and TGF-β signaling, the mechanism through which Pb regulates mesenchymal cell fate determination is complex and likely involves modulation and integration of multiple signaling pathways. Increased understanding of the mechanisms through which Pb regulates stem cell fate and subsequently affects tissue repair is critical, given the sensitivity of these tissues and the ubiquitous presence of Pb in our society and in other industrialized and developing nations.
